# Impact of bleeding and thrombosis on outcome of 945 COVID-19 VV-ECMO cases from a German registry

**DOI:** 10.3389/fmed.2025.1649217

**Published:** 2025-08-20

**Authors:** Johannes Herrmann, Janno Schade, Patrick Meybohm, Noah Paschke, Martha E. Hübsch, Quirin Notz, Julie Groene, Daniel Röder, Peter Kranke, Michaela L. Merten, Micha Landoll, Peter Spieth, Stefan Kluge, Dominik Jarczak, Kevin Roedl, Michael Sonntagbauer, Christian Putensen, Jens-Christian Schewe, Stefan F. Ehrentraut, Stefan Kreyer, Andreas Wehrfritz, Ixchel Castellanos, Karl Bihlmaier, Karsten Schmidt, Thorsten Brenner, Frank Herbstreit, Florian Espeter, Jan Wiefhoff, Richard K. Ellerkmann, Daniel Oswald, Björn Ellger, Gösta Lotz, Florian J. Raimann, Tobias Wengenmayer, Dawid L. Staudacher, Viviane Zotzmann, Onnen Moerer, Christian Kühn, Matthias Kochanek, Ralf Muellenbach, Patricia Glaser, Falk Fichtner, Marc Bodenstein, Michael Findeisen, Vanessa Rembold, Markus Heim, Gerhard Schneider, Tobias Lahmer, Jan-Sören Padberg, Carsten Hullermann, Philipp M. Lepper, Andre P. Becker, Guy Danziger, Carlos Metz, Peter Rosenberger, Valbona Mirakaj, Alice-Marie Bernard, Stephan Braune, Rebecca Roth, Anna Grau, Peter Heuschmann, Christian Karagiannidis, Christopher Lotz

**Affiliations:** ^1^Department of Anesthesiology, Intensive Care, Emergency and Pain Medicine, University Hospital Würzburg, Würzburg, Germany; ^2^Department of Pneumology and Critical Care Medicine, Cologne-Merheim Hospital, ARDS and ECMO Center, Kliniken der Stadt Köln, Witten/Herdecke University Hospital, Cologne, Germany; ^3^Department of Cardiovascular Engineering, Institute of Applied Medical Engineering, Medical Faculty, RWTH Aachen University, Aachen, Germany; ^4^Institute for Computational Biomedicine, Medical Faculty, RWTH Aachen University, Aachen, Germany; ^5^Department of Anesthesiology and Intensive Care Medicine, University Hospital, Dresden at the TU Dresden, Dresden, Germany; ^6^Department of Intensive Care, University Medical Center Hamburg-Eppendorf, Hamburg, Germany; ^7^Department of Anesthesiology and Intensive Care Medicine, University Hospital Bonn, Bonn, Germany; ^8^Department of Anaesthesiology, Intensive Care Medicine and Pain Therapy, University Medical Centre Rostock, Rostock, Germany; ^9^Department of Anaesthesiology, University Hospital Erlangen, Friedrich-Alexander University, Erlangen- Nuernberg (FAU), Erlangen, Germany; ^10^Department of Internal Medicine 4 – Nephrology and Hypertension, University Hospital Erlangen, Friedrich-Alexander University, Erlangen-Nuernberg (FAU), Erlangen, Germany; ^11^Department of Anesthesiology and Intensive Care Medicine, University Hospital Essen, University Duisburg-Essen, Essen, Germany; ^12^Department of Anesthesiologie and Intensive Care Medicine, Klinikum Dortmund, Klinikum University Witten/Herdecke, Dortmund, Germany; ^13^Department of Anesthesiology, Intensive Care Medicine and Pain Therapy, Clinic Centre Westfalen, Dortmund, Germany; ^14^Department of Anesthesiology, Intensive Care Medicine and Pain Therapy, Goethe University Frankfurt, University Hospital Frankfurt, Frankfurt, Germany; ^15^Department of Cardiology and Angiology I (Heart Center Freiburg - Bad Krozingen), Medical Center – University of Freiburg, Faculty of Medicine, University of Freiburg, Freiburg, Germany; ^16^Interdisciplinary Medical Intensive Care (IMIT), Medical Center - University of Freiburg, Faculty of Medicine, University of Freiburg, Freiburg, Germany; ^17^Ortenau Klinikum, Department of Cardiology, Pneumology, Acut Geriatrics, Angiology, Intensiv Care Medicine and Thoracic Surgery (DKPAAIT), Offenburg, Germany; ^18^Department of Anesthesiology, University Medical Center Göttingen, Göttingen, Germany; ^19^Department of Cardiothoracic, Transplanatation and Vascular Surgery, Hannover Medical School, Hannover, Germany; ^20^Center for Integrated Oncology Aachen Bonn Cologne Duesseldorf, Department I of Internal Medicine, Faculty of Medicine and University Hospital Cologne, University of Cologne, Cologne, Germany; ^21^Department of Anesthesiology and Critical Care Medicine, ARDS/ECMO-Center, Campus Kassel of the University of Southampton, Kassel, Germany; ^22^Departement of Anaesthesia, perioperative Medicine and Interdisciplinary Intensive Care Medicine, ECLS/ECMO-Center, Asklepios Klinik Langen, Langen, Germany; ^23^Department of Anesthesiology and Intensive Care Medicine, University of Leipzig Medical Center, Leipzig, Germany; ^24^Department of Anesthesiology, University Medical Center of the Johannes Gutenberg-University Mainz, Mainz, Germany; ^25^Klinik für Pneumologie, Internistische Intensiv- und Beatmungsmedizin, München Klinik Harlaching, Munich, Germany; ^26^Department of Anaesthesiology and Intensive Care Medicine, Klinikum Rechts der Isar, TUM School of Medicine and Health, Munich, Germany; ^27^Department of Internal Medicine II, University Hospital Rechts der Isar, School of Medicine, University of Munich, Munich, Germany; ^28^Department of Cardiology I – Coronary and Peripheral Vascular Disease, Heart Failure, University Hospital Muenster, Muenster, Germany; ^29^Department of Internal Medicine V- Pneumology, Allergology and Critical Care Medicine, Saarland University, Homburg, Germany; ^30^Department of Anesthesiology and Intensive Care Medicine, University Hospital Tübingen, Eberhard Karls University Tübingen, Tübingen, Germany; ^31^Department of Medical Intensive Care and Emergency Medicine, St. Franziskus-Hospital Muenster, Münster, Germany; ^32^Institute of Clinical Epidemiology and Biometry, University of Würzburg, Würzburg, Germany; ^33^Clinical Trial Center Würzburg, University Hospital Würzburg, Würzburg, Germany; ^34^Department of Health Services Research, German Cancer Society, Berlin, Germany; ^35^Institute for Medical Data Science, University Hospital Würzburg, Würzburg, Germany

**Keywords:** COVID-19, acute respiraratory distress syndrome, extracorporeal membrance oxygenation, bleeding, thromboembolism

## Abstract

Bleeding and thromboembolic events (BTE) increase the mortality of COVID-19 acute respiratory distress syndrome (ARDS) treated with extracorporeal membrane oxygenation (ECMO). The current analysis aimed to assess frequency and determinants of BTE according to their location and severity in a retrospective analysis of the German ECMO COVID-19 registry. Logistic regression was applied to identify factors influencing ICU survival as well as variables associated with risks of BTE. In total, 708 of 945 patients (75%) suffered from BTE. Overall, 1,348 events were registered, including 406 (30%) major bleeding and 258 (19%) major thromboembolic events. Most common major bleeding locations were intracranial (*n* = 133, 10%) and pulmonary bleeding (*n* = 116, 9%). In-ICU survival was 35, 46% without BTE and 22% with major bleeding (*p* < 0.05). In summary, major bleeding was a core outcome-determinant of COVID-19 ECMO mortality with intracranial major bleeding as the most devastating complication (OR: 5.3; CI: 2.9–9.9; *p* < 0.001). Neither major thromboembolism nor minor BTE impacted ICU-mortality. Potentially modifiable factors associated with major bleeding included prolonged duration of ECMO >14 days (OR: 2.9; CI 1.8–4.7; *p* < 0.001) and platelet counts <100.000/μL ≥ 72 h (OR: 2.0; CI 1.1–3.6; *p* = 0.018). Hence, prevention, early recognition and treatment of major bleedings are key to increase the survival of COVID-19 ECMO. In this regard, our data indicate that the implementation of early weaning strategies to minimize duration of ECMO therapy and prevention of prolonged thrombocytopenia with platelet counts <100.000/μl ≥ 72 h could decrease the risk of devastating bleeds and could ameliorate survival.

**Clinical trial registration:**

Registered in the German Clinical Trials Register (study ID: DRKS00022964), retrospectively registered, September 7th 2020, https://drks.de/DRKS00022964.

## Graphical abstract

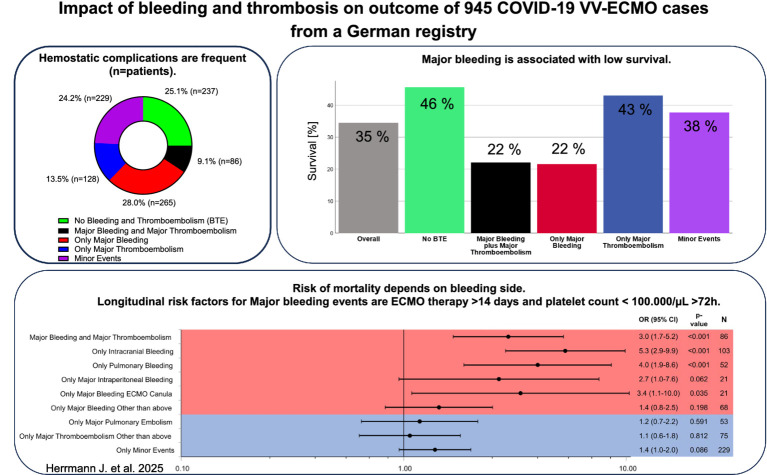

## Background

Veno-venous extracorporeal membrane oxygenation (V-V ECMO) serves as a bridge to recovery in severe COVID-19 induced acute respiratory distress syndrome (ARDS) (1). In-ICU mortality of COVID-19 ECMO ranges from 31 to 46%, whereas bleeding complications represent an independent risk factor of non-survival (2, 3).

Bleeding and thromboembolism are clinical manifestations of deranged hemostasis. Alterations are associated with the ARDS as well as ECMO associated coagulopathy (4, 5). ECMO associated coagulopathy is characterized by platelet dysfunction, an acquired von Willebrand syndrome (vWF) (6), consumption of coagulation factors, as well as increased prothrombin fragment 1.2, thrombin-antithrombin complex, and D-dimers (7). In addition, the large foreign surface area of the ECMO circuit requires systemic anticoagulation, which can aggravate the consumptive coagulopathy. COVID-19 itself may also worsen the situation, as the viral disease induces a coagulopathy mainly predisposing to thrombotic complications (8).

A previous study found evidence for venous thromboembolism in 31%, arterial emboli in 5% and pulmonary embolism in 19% in COVID-19 patients treated on an intensive care unit (ICU) (9). A recent meta-analysis conducted in the US, Europe and Asia described bleeding complications in 7.8% and major bleeding events in 3.9% in >18,000 hospitalized COVID-19 patients (10). Incidences of BTE in severely-ill COVID-19 ECMO patients are much higher. A cohort study from the French national ECMOSARS registry found bleeding complications in 29% associated with an approximately threefold increased risk of non-survival (11). In a preceding analysis of the German nationwide ECMO COVID-19 register, major bleeding or thromboembolic events were associated with a higher probability of non-survival during ICU stay (3). Nevertheless, BTE during COVID-19 ECMO vary in severity and minor events might not be as outcome relevant as major events such as intracerebral bleeding or pulmonary embolism.

The current analysis aimed to assess frequency and determinants of bleeding and thromboembolic events according to their location and severity as well as their impact on early outcome during ICU stay in a large multicenter register study from Germany.

## Methods

### Study design and patient population

The **German COVID-19 ECMO registry** is a nation-wide retrospective observational multi-center study (3). Consecutive patients with SARS-CoV-2 induced ARDS treated with ECMO at 29 ECMO centers across Germany between January 1st 2020 and July 31st 2021 were included.

### Data collection and outcomes

The treating physicians documented anonymous data from routine clinical care in a standardized electronic case report form (RedCap®, hosted at University of Wuerzburg). Data comprise patient-related risk factors, laboratory values, ECMO runs, therapeutic regimens, complications and outcome. Patient outcomes included survival to ICU discharge, length of ECMO and ICU stay, occurrence and location of major and minor bleeding events, occurrence and location of major and minor thromboembolic events. Major bleeding events were defined according to the definition by Schulman as (1) a fatal bleeding and/or, (2) symptomatic bleeding in a critical area or organ, such as intracranial, intraspinal, intraocular, retroperitoneal, intra-articular or pericardial, or intramuscular with compartment syndrome and/or, (3) bleeding causing a fall in hemoglobin level of 20 g/L or more, or leading to transfusion of two or more units of whole blood or red cells (12). Minor bleeding events (clinical relevant non-major bleeding) were defined according to the definition by Kaatz as any sign or symptom of hemorrhage (e.g., more bleeding than would be expected for a clinical circumstance, including bleeding found by imaging alone) that does not fit the criteria for the definition of major bleeding but does meet at least one of the following criteria: (1) requiring medical intervention by a healthcare professional, (2) leading to increased level of care, (3) prompting a face to face evaluation (13). Thromboembolic events were included if diagnosed by standardized diagnostic procedures according to clinical in-house routine such as ultrasound examinations or CT scans. Documented major thromboembolic events included pulmonary thromboembolism, systemic arterial thromboembolism/ischemia and deep vein thrombosis. Diagnosis of clot formation within the ECMO circuits was at the discretion of the respective centers according to their in-house standards. According to the occurrence of BTE during ICU stay, each patient was categorized into one of the five following main groups: Major bleeding plus major thromboembolism, only major bleeding, only major thromboembolism, minor events or no BTE (serving as reference cohort). To further investigate distinct entities of BTE, each patient was categorized into the following subgroups: Major bleeding plus major thromboembolism, only major intracranial bleeding, only major pulmonary bleeding, only major intraperitoneal bleeding, only major bleeding ECMO cannula, only major bleeding other than above, only major pulmonary embolism, only major thromboembolism other than above, minor events or no BTE (serving as reference cohort).

### Statistical analysis

Descriptive statistics are expressed as median (IQR) for continuous variables and as frequencies for categorical variables (including a category for missing data). Differences between groups were tested using the Kruskal-Wallis-test (continuous variables) or χ2 test (categorical variables). χ2 test with *post hoc* pairwise z-tests were applied to analyze survival in the subgroups (major bleeding plus major thromboembolism, only major bleeding, only major thromboembolism, only minor events or no BTE). No BTE served as reference cohort. To further investigate ICU-mortality and estimated odds ratios (ORs) with corresponding 95% confidence intervals (CIs) in these distinct entities, we used unadjusted logistic regression analyses. We performed adjusted logistic regression analyses in subgroups consisting of >40 patients and performed univariate χ2 test in subgroups consisting of ≤40 patients to study estimated odds ratios (ORs) with corresponding 95% confidence intervals (CIs). Variables were selected *a priori* based on clinical background knowledge: age (41–70 or >70 years vs. 18–40 years), sex (female vs. male), duration of ECMO therapy (> 14 days vs. ≤ 14 days), blood flow (>4.5 L/min ≥ 72 h vs. ≤4.5 L/min for ≥72 h or >4.5 L/min for ≤72 h), platelet count (<100.000/μL for ≥72 h vs. ≥100.000 μL for ≥72 h or <100.000/μL for <72 h), C-reactive protein (CRP) levels (>60 mg/dL for ≥72 h vs. ≤60 mg/dL for ≥72 h or >60 mg/dL for <72 h), interleukin-6 (IL-6) (>500 pg./dL for ≥72 h vs. ≤500 pg./dL for ≥72 h or >500 pg./dL for <72 h). Data analysis was performed with SPSS Statistics version 28.0.0.1 (IBM, Armonk, USA). The significance level was set to 0.05. As this is an exploratory study, *p*-values (two-tailed) are not corrected for multiple testing and interpreted descriptively.

### Ethics

This study has been performed in accordance with the Declaration of Helsinki. The Ethics Committee of the Medical Faculty of the Julius-Maximilians-University of Wuerzburg approved the study protocol (131/20-me). Each participating ECMO centers obtained additional votes from their local ethics committee. According to German legislation, no informed consent for collecting retrospective, anonymous data is required. Hence, the Ethics Committee of the Medical Faculty of the Julius-Maximilians-University of Wuerzburg waived informed consent. This study was registered in the German Clinical Trials Register (study ID: DRKS00022964, retrospectively registered, September 7th 2020^1^).

## Results

Key results are given as graphical abstract.

### Patient population

**Figure 1:**
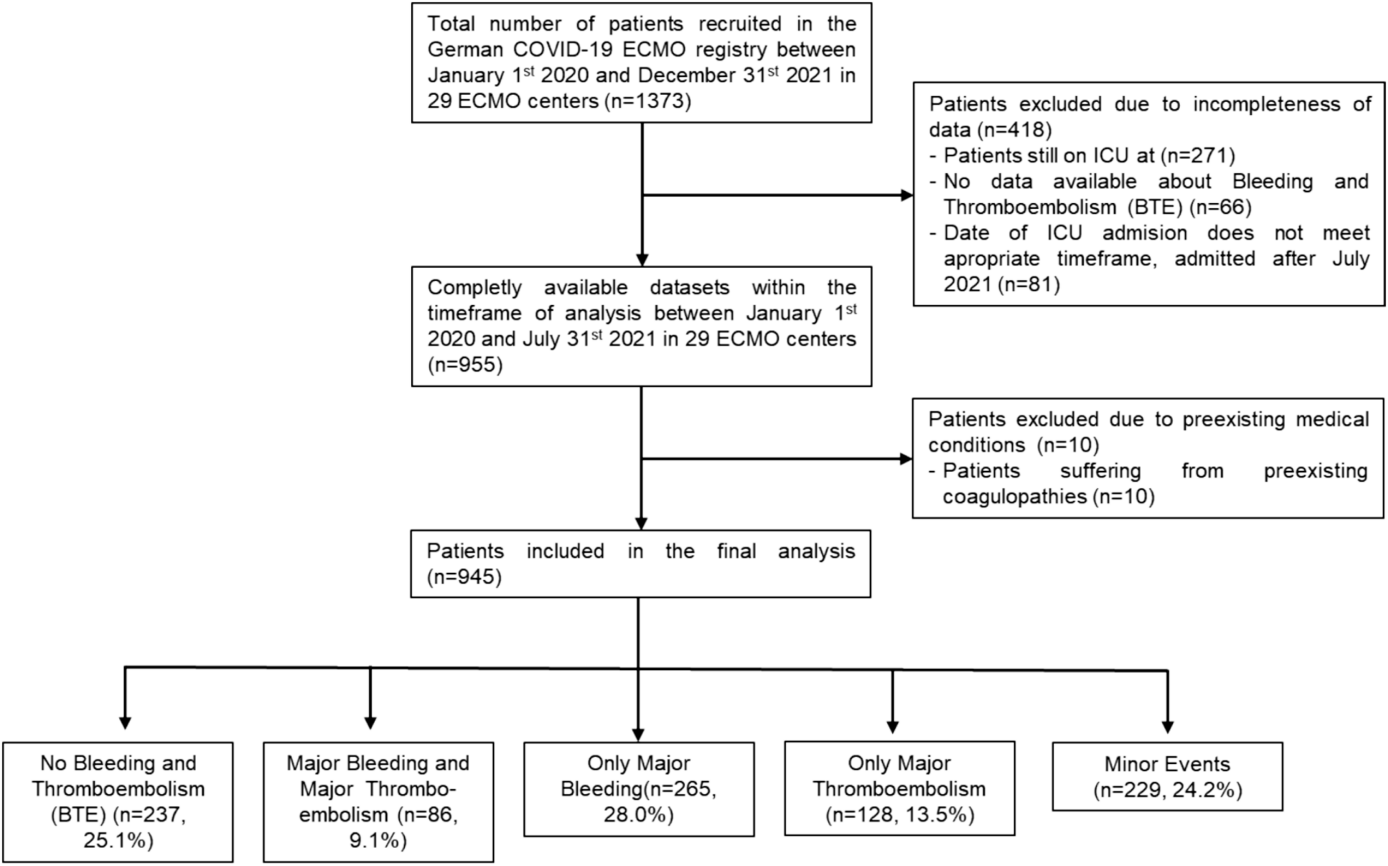
Patient recruitment. Patient recruitment is depicted in this flowchart. Number and reason for exclusion are given. Nine hundred and forty-five patients were finally included and each patient was categorized into one of the five following main groups: No Bleeding and Thromboembolism, Major Bleeding and Major Thromboembolism, Only Major Bleeding or Only Major Thromboembolism.

Between January 1^st^ 2020 and December 31^st^ 2021, 1,373 patients were entered into the database and 945 patients with complete datasets were included in the current analysis (Figure 1). Reasons for exclusion in the current analysis were: 418 datasets were incomplete (271 patients were still treated on or not discharged from the ICU after July 31^st^ 2021, respectively; 81 patients were excluded due to ICU stays outside of the defined timeframe of the analysis; in 66 patients, data on bleeding or thromboembolic events were missing). Preexisting coagulopathies excluded ten patients.

Table 1 and Table S1 provide a detailed overview on the characteristics, co-morbidities, as well as ECMO support and adjunct therapies. Most patients (85.6%) were between 41–70 years old. 735 patients (78.4%) were male. Median BMI was 29.9 (IQR 27.1–35.2). V-V ECMO used in 914 patients (96.7%) with a median duration of 333.3 h (IQR 164–576 h). Median duration of ICU care was 26 days (IQR 16–42 days). Only 36 patients (3.8%) were on long-term anticoagulation prior to ICU admission. Anticoagulation regimen on day 1 of ECMO support was unfractionated heparin in 793 (85.7%), low-molecular-weight-heparin in 45 (4.9%) or argatroban in 81 (8.8%) (data not shown).

**Table 1:** Clinical characteristics.

	Level	Overall	No BTE	Major bleeding plus thromboembolism	Only major bleeding	Only major thromboembolism	Minor events	*p*-value
*n* (%)		945 (100)	237 (25.1)	86 (9.1)	265 (28.0)	128 (13.5)	229 (24.2)	
Demographics								
Age group [years]	*n* (%)							**<0.001**
	19–40	86 (9.1%)	23 (9.7%)	7 (8.1%)	18 (6.8%)	11 (8.6%)	27 (11.8%)	
	41–70	809 (85.6%)	206 (86.9%)	67 (77.9%)	234 (88.3%)	111 (86.7%)	191 (83.4%)	
	71–80	47 (5.0%)	8 (3.4%)	11 (12.9%)	12 (4.5%)	5 (3.9%)	11 (4.8%)	
	Missing	3	0	1	1	1	0	
Sex (by birth)	*n* (%)							**0.049**
	Male	735 (78.4%)	181 (76.7%)	77 (90.6%)	209 (79.5%)	94 (74.6%)	174 (76.7%)	
	Female	202 (21.6%)	55 (23.3%)	8 (9.4%)	54 (20.5%)	32 (25.4%)	53 (23.3%)	
	Missing	8	1	1	2	2	2	
BMI [kg/cm^2^]	Median (IQR)	29.9 (27.1–35.2)	31.0 (27.2–36.5)	28.2 (26.1–31.0)	29.6 (26.9–35.1)	29.6 (27.6–35.0)	30.9 (27.7–36.9)	**<0.001**
	Missing	24	7	2	8	4	3	
ECMO								
Mode of ECMO support	*n* (%)							**0.017**
	V-V ECMO	914 (96.7%)	236 (99.6%)	79 (91.9%)	257 (97.0%)	121 (94.5%)	221 (96.5%)	
	V-A ECMO	19 (2.0%)	1 (0.4%)	3 (3.5%)	6 (2.3%)	5 (3.9%)	4 (1.7%)	
	V-VA ECMO	12 (1.3%)	0 (0.0%)	4 (4.7%)	2 (0.8%)	2 (1.6%)	4 (1.7%)	
	Missing	0	0	0	0	0	0	
Mean duration of ECMO therapy [h]	Median (IQR)	333.3 (164–576)	216 (108–426)	356 (168–761)	379 (175.5–648)	355 (190–561)	384 (192–597)	**<0.001**
	Missing	3	0	0	2	0	1	
Outcome								
Mean duration of ICU stay [d]	Median (IQR)	26 (16–42)	21 (14–37)	27 (15–50)	27 (17–41)	28 (17.5–44.5)	28 (19–45)	**0.004**
	Missing	45	11	3	14	4	13	
Mean duration of hospital stay [d]	Median (IQR)	29 (18–45)	25 (17–41.5)	27 (15–51)	30 (19.5–44.5)	29.5 (20–50)	32 (21–48)	**0.015**
	Missing	84	17	7	25	8	27	
In-ICU survival	*n* (%)	325 (34.5%)	108 (45.6%)	19 (22.1%)	57 (21.6%)	55 (43.0%)	86 (37.7)	**<0.001**
	Missing	2	0	0	1	0	1	

Clinical characteristics are presented in total population and in subgroups no BTE vs. major bleeding plus major thromboembolism vs. only major bleeding vs. only major thromboembolism vs. minor events, respectively. Descriptive statistics are expressed as frequencies for categorical variables (including a category for missing data) and as median (IQR) for continuous variables. Differences between groups were tested using the Kruskal-Wallis-test (continuous variables) or Pearson-*χ*2-test (categorical variables). *P*-values less than 0.05 were considered as statistically significant.

Significant *P*-values are given in bold values.

### Bleeding and thromboembolic events

We found 1,348 BTE in 945 patients: 406 major bleeding events (1 event in 303 patients, 2 events in 42 patients, ≥3 events in 6 patients), 506 minor bleeding events (1 event in 305 patients, 2 events in 69 patients, ≥3 events in 20 patients), 258 major thromboembolic events (1 event in 180 patients, 2 events in 28 patients, ≥3 events in 6 patients) and 178 minor thromboembolic events (1 event in 106 patients, 2 events in 17 patients, ≥3 events in 10 patients) (Table 2). Two hundred and thirty-seven patients (25.1%) had neither a bleeding nor a thromboembolic event. Major bleeding as the only event occurred in 265 patients (28.0%). Eighty-six patients (9.1%) had major bleeding and major thromboembolic events. Hundred twenty-eight patients (13.5%) solely had major thromboembolic events. Two hundred twenty-nine patients (24.2%) solely had minor bleeding or minor thromboembolic events.

**Table 2:** Total number and locations of bleeding and thromboembolic events.

Entity and location	*N* events	*N* patients	% of total events	% of total patients (*N* = 945)
**Overall**	**1,348**	**708**	**100**	**74.9**
**Major bleeding event**	**406**	**346**	**30.1**	**36.6**
Intracranial	133	133	9.9	14.1
Pulmonary	116	105	8.6	11.1
Intraperitoneal	42	38	3.1	4.0
ECMO cannula	33	33	2.4	3.5
Surgical site or catheter	25	24	1.9	2.5
Mouth	25	25	1.9	2.6
Retroperitoneal	10	10	0.7	1.1
Heart	5	4	0.4	0.4
Bladder	5	5	0.4	0.5
Nose	6	6	0.4	0.6
Muscle (with compartment syndrome)	4	4	0.3	0.4
Spine	1	1	0.1	0.1
Eye	0	0	0	0
Joint	0	0	0	0
Missing	1	1	0.1	0.1
**Minor bleeding event**	**506**	**410**	**37.5**	**43.4**
ECMO cannula	141	140	10.5	14.8
Nose	95	94	7.0	9.9
Mouth	94	94	7.0	9.9
Surgical site or catheter	72	67	5.3	7.1
Intraperitoneal	38	37	2.8	3.9
Bladder	30	30	2.2	3.2
Skin	19	18	1.4	1.9
Muscle (without compartment syndrome)	9	8	0.7	0.8
Missing	8	8	0.6	0.8
**Major thromboembolic event**	**258**	**128**	**19.1**	**13.5**
Pulmonary embolism	115	113	8.5	12.0
Systemic arterial embolus or ischemia	80	72	5.9	7.6
Peripheral vessel	34	34	2.5	3.6
Ischemic stroke	14	14	1.0	1.5
Myocardial infarction	9	9	0.7	1.0
Mesenteries	10	10	0.7	1.1
Liver	5	5	0.4	0.5
Spleen	6	6	0.4	0.6
Kidney	1	1	0.1	0.1
Deep vein thrombosis	63	56	4.7	5.9
**Minor thromboembolic event**	**178**	**133**	**13.2**	**14.1**
Oxygenator clot	108	71	8.0	7.5
Other	70	68	5.2	7.2

Total number and locations of bleeding and thromboembolic events, as well as the respective frequencies in relation to the number of total events are shown. Multiple mentioning of events in terms of different entities or repetitive events in one single patient are possible.

Results from main groups are given in bold.

The most common major bleeding locations were intracranial hemorrhage and pulmonary bleeding. Hundred and ten patients (11.7%) exclusively suffered from intracranial hemorrhage without additional BTE. Pulmonary embolism was the most common thromboembolic event, followed by clotting of the ECMO oxygenator.

### Blood products

Application and dosage of blood products are depicted in Supplementary Table S1. Patients suffering from only mayor bleeding received the highest volume with median of 4,500 mL (IQR 2150–8,000) packed red blood cells (PRBC), 800 mL (IQR 200–2,300) platelet concentrates (PC) and 880 mL (IQR 0-2200 mL) fresh frozen plasma (FFP). Patients with no BTE received the lowest volume of blood products (PRBC: 1500 mL (IQR 600–2,775), PC: 0 mL [IQR 0–400), FFP 0 mL (IQR 0-400 mL)], respectively. Prothrombin complex concentrate (PCC) was used in 109 patients (11.6%). Patients with major bleeding plus thromboembolism received PCC in 20.2% (cumulative dose: 3000 IU, IQR: 2500–6,500), patients with only major bleeding in 22.5% (cumulative dose: 3000 IU, IQR: 2000–4,000). Coagulation factor XIII was used in 342 patients (36.5%), most frequently in patients with only major bleeding (50.8%) with a cumulative dose of 3,750 IU (IQR: 3000–5,000). A quarter of patients received antithrombin supplementation. Fibrinogen was administered in 158 patients (16.8%), coagulation factor VII only in 7 patients (0.8%) (data not shown).

### Outcome

**Figure 2:**
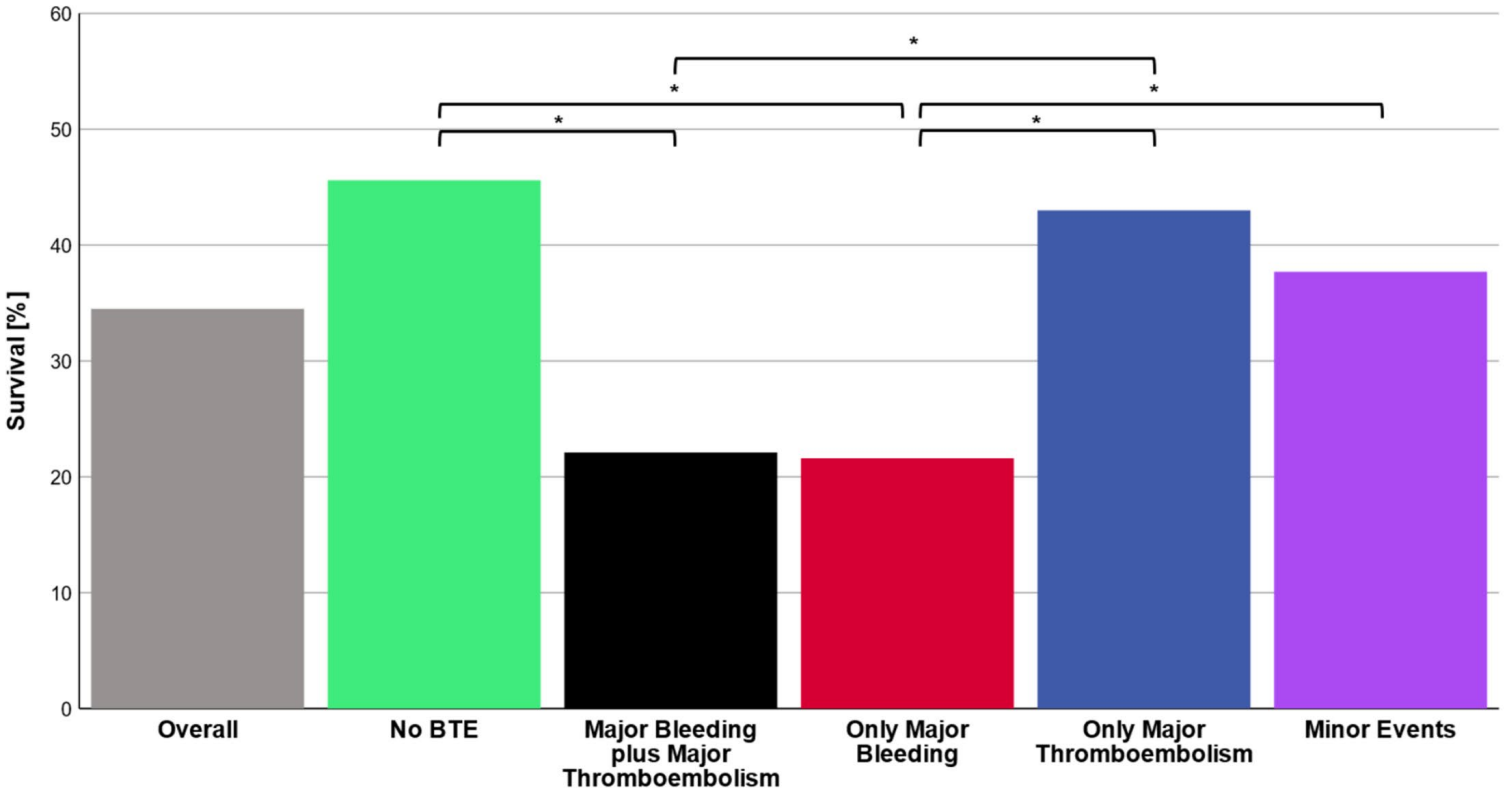
Survival. Total in-ICU survival rates are presented for the overall cohort as well as for the main groups no bleeding or thromboembolism (no BTE), major bleeding plus major thromboembolism, only major bleeding, only major thromboembolism and minor events, respectively. χ2 test with *post hoc* pairwise z-test was applied to analyze survival in the subgroups. *p*-values less than 0.05 were considered as statistically significant (*).

**Figure 3:**
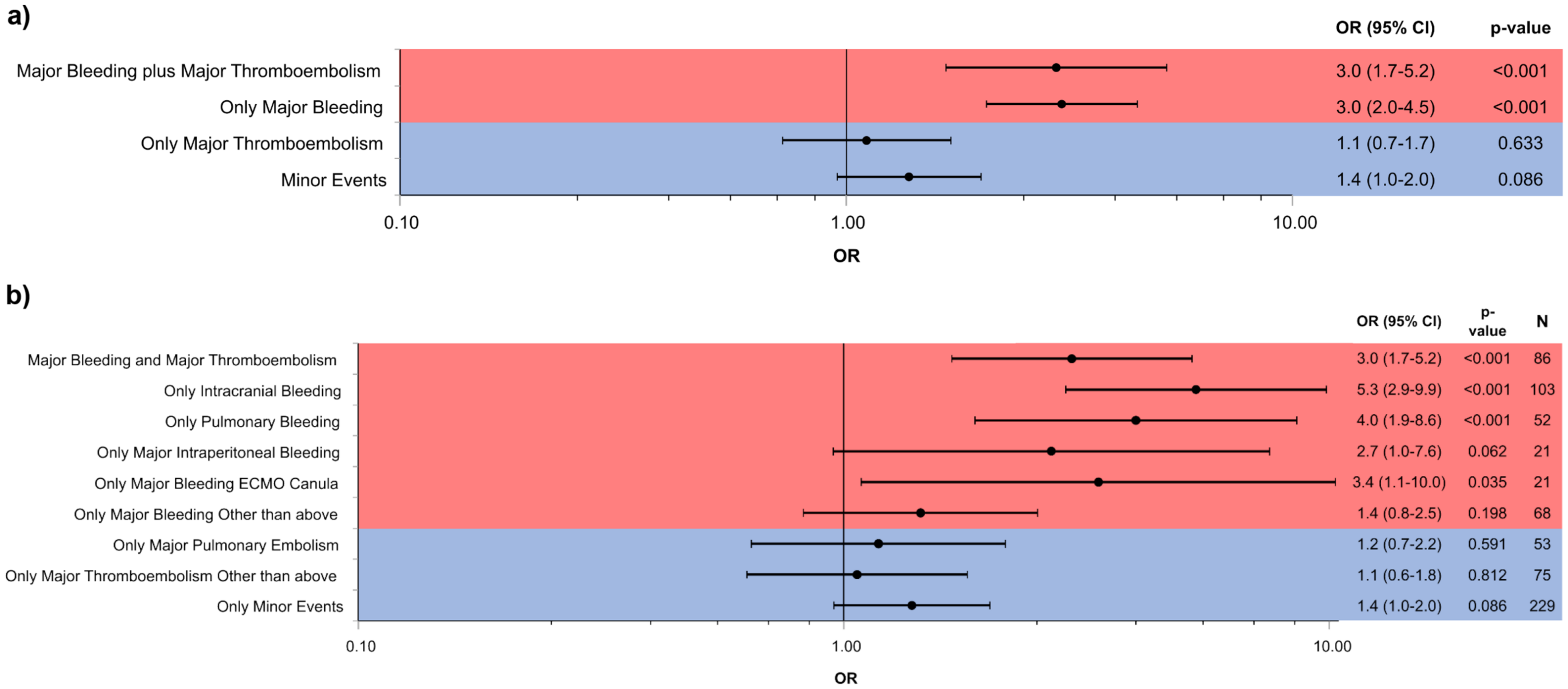
Subgroup specific risk for in-ICU mortality. Subgroup specific risk for in-ICU mortality in **(a)** main groups and in **(b)** specific subgroups are depicted. **(a)** Each patient from the cohort was categorized into one of the five following main groups: Major bleeding plus major Thromboembolism, only major bleeding, only major thromboembolism, minor events, no BTE (serving as reference cohort). **(b)** In a second step, we investigated specific risk for in-ICU mortality in distinct entities. Each patient from the cohort was categorized into one of the subgroups: Major bleeding plus major thromboembolism, only major intracranial bleeding, only major pulmonary bleeding, only major intraperitoneal bleeding, only major bleeding ECMO cannula, only major bleeding other than above, only major pulmonary embolism, only major thromboembolism other than above, minor Events, no BTE (serving as reference cohort). Analyses were performed by unadjusted logistic regression. OR, CI, *p*-values and number of patients in subgroups are given. *p*-values less than 0.05 were considered as statistically significant.

Overall survival to ICU discharge was 34.5% (325/945 patients) (Figure 2). Survival significantly differed between patient groups defined by type of BTE (*p* < 0.001). In patients without any bleeding or thromboembolic event survival was 45.6%. Survival in patients with only a major thromboembolic event was 43.0 and 37.7% in patients with only a minor bleeding or thromboembolic event. Patients with pulmonary embolism as the sole major BTE survived in 42.2% of the cases. Only major thromboembolism (OR 1.1 [0.7–1.7; *p* = 0.633]) or minor events (OR 1.4 [1.0–2.0; *p* = 0.086]) were not significantly associated with non-survival compared to no BTE. Survival in patients with a major bleeding event was 21.6% with an odds ratio (OR) of 3.0 [2.0–4.5; *p* < 0.001] for non-survival versus no BTE and 22.1% in patients with a major bleeding plus major thromboembolic event with an OR of 3.0 [1.7–5.2; *p* < 0.001] (Figure 3A). Survival in patients with intracranial bleeding was 12% with an OR for non-survival of 5.3 [2.9–9.9; *p* < 0.001]. Furthermore, the OR for non-survival was 4.0 [1.9–8.6; *p* < 0.001] for only pulmonary bleeding and 3.4 [1.1–10.0; *p* = 0.035] in case of only major bleeding ECMO cannula compared to no BTE (Figure 3B). *Post hoc* analyses revealed significantly lower survival in patients with major bleeding and major bleeding plus major thromboembolic compared to no BTE (*p* < 0.05, respectively) or major thromboembolism (*p* < 0.05, respectively).

### Risk factors

**Figure 4:**
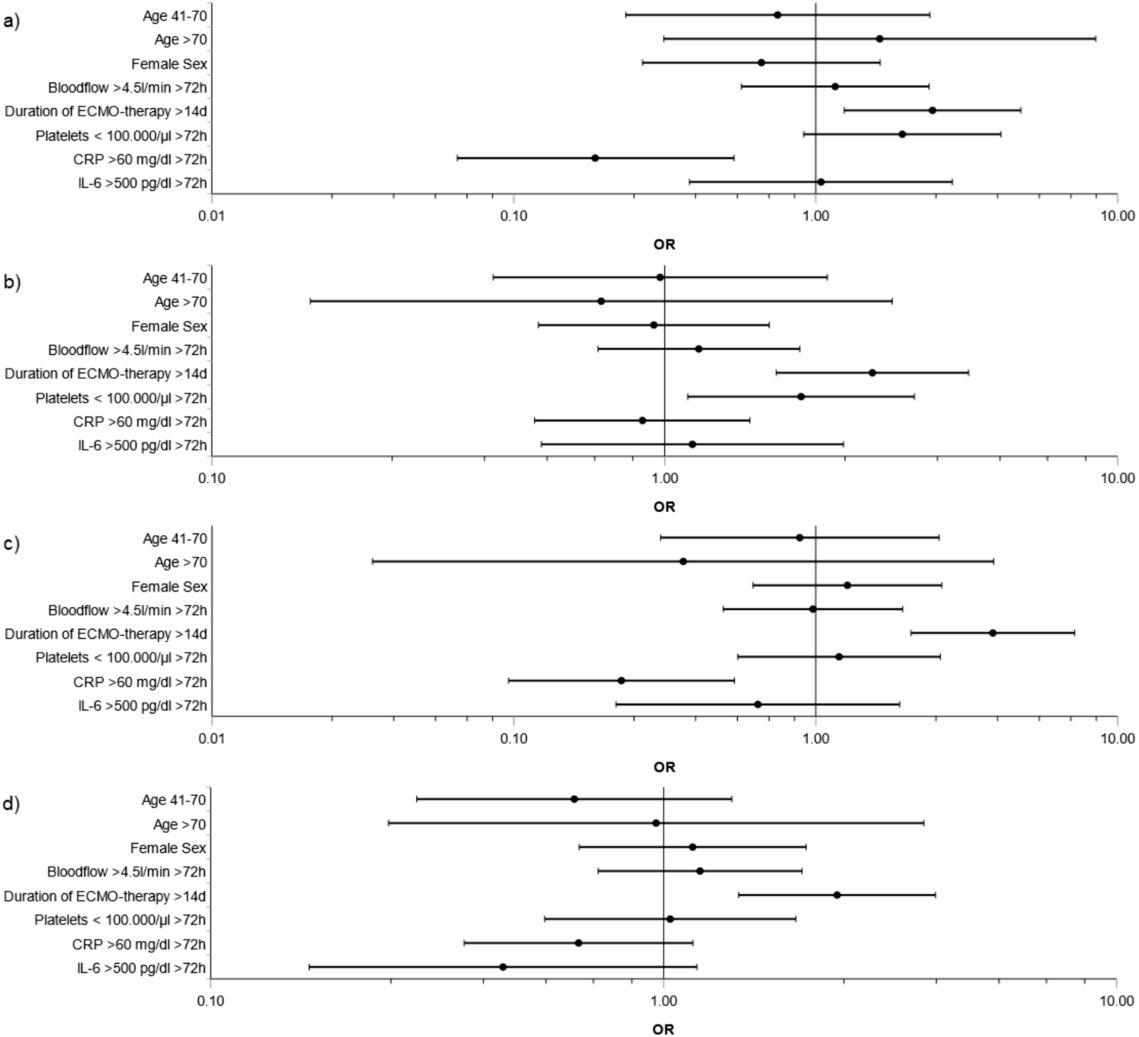
Risk factors for BTE during ECMO support in main groups. Subdivisions show risk factors during ECMO support for the main groups **(a)** Major bleeding plus thromboembolism, **(b)** Major bleeding, **(c)** Major thromboembolism, **(d)** Minor events. Variables were selected *a priori* based on clinical background knowledge: age (41–70 or >70 years vs. 18–40 years), sex (female vs. male), duration of ECMO therapy (>14 days vs. ≤14 days), blood flow (>4.5 L/min ≥ 72 h vs. ≤4.5 L/min for ≥72 h or >4.5 L/min for ≤72 h), platelet count (<100.000/μL for ≥72 h vs. ≥100.000 μL for ≥72 h or <100.000/μL for <72 h), C-reactive protein (CRP) levels (>60 mg/dL for ≥72 h vs. ≤60 mg/dL for ≥72 h or >60 mg/dL for <72 h), interleukin-6 (IL-6) (>500 pg./dL for ≥72 h vs. ≤500 pg./dL for ≥72 h or >500 pg./dL for <72 h). Analysis was performed by adjusted logistic regression. OR and CI are depicted.

Factors associated with major bleeding were duration of ECMO therapy >14 days (OR: 2.9; CI 1.8–4.7; *p* < 0.001; reference: ≤14 days) and platelet count <100.000/μL for ≥72 h (OR: 2.0; CI 1.1–3.6; *p* = 0.018; reference: ≥100.000 μL for ≥72 h or <100.000/μL for <72 h). In addition, duration of ECMO therapy >14 days was associated with major bleeding and thromboembolism (OR: 2.4; CI 1.2–4.8; *p* = 0.01), only major thromboembolism (OR: 3.9; CI 2.1–7.2; *p* < 0.001) and minor events (OR: 2.4; CI 1.5–4.0; p < 0.001) (respective reference: duration of ECMO ≤14 days). In contrast, CRP level >60 mg/dL for ≥72 h was associated with lower risk for major bleeding and thromboembolism (OR: 0.2; CI 0.07–0.5; *p* = 0.002), and major thromboembolism (OR: 0.2; CI 0.1–0.5; *p* < 0.001) compared to reference CRP ≤ 60 mg/dL for ≥72 h or >60 mg/dL for <72 h (Figures 4A–D).

Considering distinct bleeding and thromboembolic entities, prolonged ECMO therapy >14 days was associated with only pulmonary bleeding (OR: 4.4; CI 1.9–10.3; *p* < 0.001) compared to reference duration of ECMO ≤14 days. Intracranial bleeding only (OR: 2.3, CI 1.1–4.6; *p* = 0.021) and major intraperitoneal bleeding only (OR: 19.3, CI 1.7–217.5; *p* = 0.017) were associated with platelet count <100.000/μL for ≥72 h (reference: ≥100.000 μL for ≥72 h or <100.000/μL for <72 h). OR of only major pulmonary embolism was 3.2 (CI 1.3–7.7; *p* = 0.011) for duration of ECMO >14 days (reference: ECMO ≤14 days) (Supplementary Figures S1, S2). Sensitivity analyses for the models are given in Supplementary Figures S3–S5.

## Discussion

BTE were common in COVID-19 ECMO patients. Major bleeding was the core outcome parameter associated with ICU survival. Approximately one-third of COVID-19 ECMO patients survived to ICU discharge as previously discussed in our preceding analysis of the COVID-19 ECMO database (3). One-third of the patients suffered from major bleeding, a situation in which survival halved. Odds of non-survival increased three-fold with major bleeding. The most severe complication was intracranial bleeding with five-fold increased odds of non-survival compared to patients without BTE. Thromboembolic events on the contrary did not decrease survival of COVID-19 ECMO.

Multiple studies identified bleeding complications as a factor of non-survival in COVID-19 ECMO, including our own preceding analysis (2, 3, 11). Sex was not associated with increased risk for BTE although 78% of the patients were male. In COVID-19 male sex was found to be associated with higher disease severity and case fatality (14) and multiple studies found that the majority of patients with COVID-19 ARDS and non-COVID-19 ARDS are male (15, 16). In this context, the predominance of male sex among our ECMO cohort is equal to preceding similar studies (17). A retrospective study of 620 COVID-19 ECMO patients from France found bleeding events in 29% and an odds of 2.91 for in-hospital mortality. As corroborated by our data, thrombotic events were not associated with increased in-hospital mortality (11). In our larger cohort, we were able to differentiate between major and minor bleeding, as well as major and minor thromboembolic events. We found a similar incidence for the sole occurrence of major bleeding, as well as an even higher number of minor bleeding. However, differentiation of bleeding severity and localization is critical to determine core outcomes. In this regard, a recent modified Delphi study suggested major bleeding and intracranial hemorrhage as core outcomes to assess in all ECMO research (18). Prior non-COVID-19 and COVID-19 ECMO studies often described device-related or cannulation site bleeding as the most frequent bleeding locations (11). Our data show that cannulation side bleeding is frequent, but mostly minor. In our cohort, intracranial hemorrhage most severely affected survival and was the most frequent major bleeding. We found intracranial hemorrhage in 14% of the patients and a more than five-fold increased risk of non-survival in these patients. Previous studies described incidences of 8–12% and an even higher odds ratio of non-survival (11, 18). Data on 63 ECMO autopsy cases found intracranial bleeding in 21% of the patients and classified it as the immediate or underlying cause of death in 78% (19). Besides intracranial hemorrhage, pulmonary bleeding is also frequent. Pulmonary hemorrhage is always a critical bleeding event as worsening gas exchange due to obstruction of the tracheobronchial tree, as well as continued high ECMO blood flows potentially result in a vicious cycle. The 2016 Extracorporeal Life Support Organization registry described pulmonary hemorrhage in 6.1% of non-COVID-19 ECMO (20), whereas in COVID-19 ECMO 9% of the patients had pulmonary bleeding (11). Our cohort had a similar incidence of pulmonary bleeding, which lead to a fourfold increase in odds of non-survival.

The identification of modifiable risk factors is pivotal to ameliorate core outcomes. Pathophysiological changes are complex, whereas, over-activation of the coagulation cascade and suppression of fibrinolysis (21) during COVID-19 result from hypercytokinemia, hyperinflammation, endothelial damage and platelet activation (22–24). Prior studies in non-COVID-19 ECMO already described cross-sectional data on thrombocytopenia as a risk factor of intracranial hemorrhage (25). In order to depict the dynamics of ECMO- and ICU treatment we analyzed a set of longitudinal parameters of therapy. Our granular data suggest that extended periods of low platelet counts increased the risk of major bleeding, in particular intracranial bleeding. Duration of ECMO was widely associated with major and minor bleeding and thromboembolic events, which is in line with data from the international ELSO registry in patients on ECMO previous to the COVID-19 pandemic (26). We also found that extended periods of high CRP levels were associated with lower risks for major bleeding and thromboembolism or major thromboembolism alone. CRP is a modulator of acute inflammation and processes both pro-inflammatory and anti-inflammatory properties (27). Its function in thromboinflammation appears significantly dependent upon conformational changes that can occur in response to a range of stimuli (28). Levels of CRP > 100 mg/L at initial presentation increased the risk of venous thromboemboli in COVID-19 patients, whereas severity of the thrombotic events was not specified (29). Furthermore, in prior study of 321 ECMO patients lower values of C-reactive protein associated with hemorrhage, corroborating the complex relationship between inflammation and bleeding in ECMO patients (30).

Previous reports also found a positive correlation between the incidence of venous thrombi or thrombemboli and the length of ECMO support (31, 32). Although direction of causality remains unclear, patients with any BTE had ECMO runtimes more than 1.5 times longer compared to no BTE. One third of our severely ill ECMO cohort had thrombi or thromboemboli, which is similar to the upper range of prior studies describing incidences of 13.5–31% (33, 34). Pulmonary embolism was present in 12.0% of our ECMO patients, whereas others found incidences as high as 26% in COVID-19 ICU patients (35). Concomitantly, our data suggest that major thromboembolic events have less impact onto survival compared to major bleeding. Survival in patients with sole major thromboembolic events was similar to no BTE and major thromboembolic events in conjunction with major bleeding did not worsen survival compared to only major bleeding. When considering pulmonary embolism as the sole major BTE, survival was comparable to patients without any bleeding or thromboembolic event. However, clinical features of major thromboembolism vary from intermediate to high-risk events and mechanisms of subsequent hemodynamic deterioration differ. We cannot decipher the magnitude of the pulmonary emboli and subsequent hemodynamic changes. As such, medium or high-risk events must not be underestimated.

Bleeding complications frequently cause transfusion of blood products to enhance oxygen delivery and coagulation. In the PROTECMO study, 83% of the patients received at least one unit of PRBC and a median of 1.4 liters PRBC during a median ECMO duration of 11 days (36). In our study, the amount of blood products greatly varied between groups. Patients with major bleeding received more PRBC, as well as additional blood products compared to those without any BTE. However, patients without any BTE already received a mean of 1.5 liters of PRBC and patients with only minor bleeding events more than 3 liters of PRBC. Moreover, patients with only minor bleedings received a similar amount of PCC as those with major bleeding. Factor XIII was administered to a similar amount in all groups, as previous studies described an acquired factor XIII deficiency in patients with COVID-19 (24) or non-COVID-19 caused infection and sepsis (37, 38). In particular, ARDS patients had a lower factor XIII activity, further deteriorating in a time-dependent manner after the initiation of ECMO (39). In line with this data, factor XIII activity was below normal ranges on day 1 of ECMO support across all groups in our cohort. We cannot decipher the impact of blood product administration on survival, nevertheless our data corroborate that current practice warrants a high need for blood products in any case of ECMO support.

Limitations of our study include an observation period limited to ICU treatment only without any long-term follow up. In addition, we did not document any information on the time of BTE-complication, limiting the interpretation of our findings. Hence, we can not establish a direct temporal connection between the occurrence of a bleeding complication and, e.g., PLT less than 100,000 for 72 h. Our data only allow the conclusion that PLT less than 100,000 for 72 h at any time during ICU treatment was associated with an increased risk of major bleeding. Moreover, we focused on core outcomes. We used definitions for major bleeding (“Definition of major bleeding in clinical investigations of antihemostatic medicinal products in non-surgical patients“) and minor bleeding (“Definition of clinically relevant non-major bleeding in studies of anticoagulants in atrial fibrillation and venous thromboembolic disease in non-surgical patients”) by the International Society on Thrombosis and Haemostasis (12, 13), which slightly differ from ELSO definitions. They allow detection of fatal major bleedings and identification of clinically relevant non-major bleedings, gaining more detailed information in this subgroup. Results might differ from previous data according original definitions of bleeding events. Multiple BTE in a single patient were common and the study assessed only major bleeding, major thromboembolic events or major bleeding plus major thromboembolic events. These groups may include additional minor bleeding or minor thromboembolic events. Moreover, diagnosis of clot formation within the ECMO circuits was at the discretion of the respective centers. This could potentially lead to underreporting of events with consecutive and significant differences in reporting between participating centers. We did not assess anticoagulation targets and, thus, were not able to assess if differences in anticoagulation schemes contributed to our results. However, a systemic review including V-V ECMO and venoarterial (V-A) ECMO cases without continuous systemic anticoagulation still found major bleeding in 28% (40), pinpointing toward the conclusion that the ECMO circuit itself primarily causes a bleeding diathesis independent of systemic anticoagulation. We did not assess timing and thresholds for administration of blood products. Hence, it is impossible to identify a potential association between the administration of blood products and ECMO complications or survival.

Our highly granular data indicate relevant implications for clinical management of patients suffering from COVID-19 ARDS on V-V ECMO. (1) Major bleeding events are more frequent and more harmfull than major thromboembolic events in terms of mortality. Aggressive anticoagulation regimens over a long period might provoke major bleeding and should not be administered without strict indication. Continuous reevaluation of individual anticoagulation strategy seems to be paramount. However, superiority of a specific anticoagulation agent and its favorable dosage on ECMO has not yet be demonstrated. There is tremendous need for randomized controlled trials to optimize anticoagulation strategies on ECMO (41). (2) Risk of BTE is associated with duration of ECMO therapy. Regular evaluation of respiratory status and early ECMO weaning trials seem to be reasonable to minimize length of ECMO support and complications. (3) Persistent thrombocytopenia <100.000/μL were associated with major bleeding, in particularly with devastating intracranial bleeding. In this regard, periods of thrombocytopenia should be avoided and implementation of platelet transfusion protocols could be considered in these patients. Besides the role of platelet count, further studies are in dire need to decipher the impact of ECMO-induced platelet dysfunction on BTE and its impact on survival.

## Conclusion

In summary, major bleeding was a core outcome-determinant of COVID-19 ECMO mortality with intracranial bleeding as the most devastating complication. Major thromboembolism or minor BTE did not alter the survival of COVID-19 ECMO. Hence, prevention, early recognition and treatment of major bleedings are key to increase the survival of COVID-19 ECMO. In this regard, our granular data indicate that the implementation of early ECMO weaning strategies could decrease the risk of devastating bleeds. Prolonged thrombocytopenia with platelet counts <100.000/μl should be avoided.

## Data Availability

The datasets presented in this article are not readily available because the datasets used and/or analyzed during the current study are available from the corresponding author on reasonable request. Requests to access the datasets should be directed to Herrmann_J4@ukw.de.

## Footnotes

^1^ https://drks.de/DRKS00022964
