# Supplementary material for: Impact of bleeding and thrombosis on outcome of 945 COVID-19 VV-ECMO cases from a German registry
Source: Front Med (Lausanne). 2025 Aug 20;12:1649217. doi: 10.3389/fmed.2025.1649217 (PMC12405335; doi:10.3389/fmed.2025.1649217)
Supplement: Supplementary file 1 [file Table_1.DOCX]

**Supplementary information**

**Impact of bleeding and thrombosis on outcome of 945 COVID-19 VV-ECMO cases from a German registry**

**Supplemental Table 1: Comorbidities, adjunct medication and laboratory values**

|  | **Level** | **Overall** | **No BTE** | **Major bleeding plus thromboembolism** | **Only major bleeding** | **Only major thromboembolism** | **Minor events** | **P-value** |
| --- | --- | --- | --- | --- | --- | --- | --- | --- |
| n (%) |  | 945 (100) | 237 (25.1) | 86 (9.1) | 265 (28.0) | 128 (13.5) | 229 (24.2) |  |
| **Comorbidities and long-term medication** | | | | | | | | |
| Charlson Comorbidity Index (0-37) | median (IQR) | 1 (0-1) | 1 (0-1) | 0.5 (0-2) | 1 (0-2) | 0 (0-1) | 1 (0-2) | 0.250 |
|  | missing | 25 | 5 | 4 | 5 | 6 | 5 |  |
| Cardiovascular disease | n (%) |  |  |  |  |  |  | 0.294 |
|  | no | 357 (39.8%) | 86 (37.7%) | 35 (44.9%) | 92 (36.2%) | 55 (46.6%) | 89 (40.5%) |  |
|  | yes | 541 (60.2%) | 142 (62.3%) | 43 (55.1%) | 162 (63.8%) | 63 (53.4%) | 131 (59.5%) |  |
|  | missing | 47 | 9 | 8 | 11 | 10 | 9 |  |
| Chronic pulmonary disease | n (%) |  |  |  |  |  |  | 0.545 |
|  | no | 803 (86.0%) | 205 (88.0%) | 74 (88.1%) | 219 (83.9%) | 112 (88.2%) | 193 (84.3%) |  |
|  | yes | 131 (14.0%) | 28 (12.0%) | 10 (11.9%) | 42 (16.1%) | 15 (11.8%) | 36 (15.7%) |  |
|  | missing | 11 | 4 | 2 | 4 | 1 | 0 |  |
| Diabetes mellitus (with and without end organ damage) | n (%) |  |  |  |  |  |  | 0.126 |
|  | no | 693 (74.0%) | 161 (68.8%) | 68 (80.0%) | 196 (75.1%) | 101 (79.5%) | 167 (72.9%) |  |
|  | yes | 243 (26.0%) | 73 (31.2%) | 17 (20.0%) | 65 (24.9%) | 26 (20.5%) | 62 (27.1%) |  |
|  | missing | 9 | 3 | 1 | 4 | 1 | 0 |  |
| Moderate or severe kidney disease | n (%) |  |  |  |  |  |  | 0.767 |
|  | no | 891 (95.3%) | 224 (96.1%) | 81 (95.3%) | 246 (94.3%) | 123 (96.9%) | 217 (94.8%) |  |
|  | yes | 44 (4.7%) | 9 (3.9%) | 4 (4.7%) | 15 (5.7%) | 4 (3.1%) | 12 (5.2%) |  |
|  | missing | 10 | 4 | 1 | 4 | 1 | 0 |  |
| Long term anticoagulation | n (%) |  |  |  |  |  |  | 0.569 |
|  | no | 805 (85.3%) | 206 (87.3%) | 73 (84.9%) | 229 (86.4%) | 105 (82.0%) | 192 (83.8%) |  |
|  | yes | 36 (3.8%) | 5 (2.1%) | 4 (4.7%) | 13 (4.9%) | 4 (3.1%) | 10 (4.4%) |  |
|  | unknown | 103 (10.9%) | 25 (10.6%) | 9 (10.5%) | 23 (8.7%) | 19 (14.8%) | 27 (11.8%) |  |
|  | missing | 1 | 1 | 0 | 0 | 0 | 0 |  |
| Long term platelet aggregation inhibitors | n (%) |  |  |  |  |  |  | **0.035** |
|  | no | 777 (82.3%) | 199 (84.0%) | 70 (81.4%) | 228 (86.0%) | 103 (80.5%) | 177 (77.6%) |  |
|  | yes | 67 (7.1%) | 15 (6.3%) | 9 (10.5%) | 15 (5.7%) | 4 (3.1%) | 24 (10.5%) |  |
|  | unknown | 100 (10.6%) | 23 (9.7%) | 7 (8.1%) | 22 (8.3%) | 21 (16.4%) | 27 (11.8%) |  |
|  | missing | 1 | 0 | 0 | 0 | 0 | 1 |  |
| **Administration of blood products and coagulation factors** | | | | | | | | |
| Packed red blood cells | n (%) |  |  |  |  |  |  | **<0.001** |
|  | no | 29 (3.2%) | 18 (8.5%) | 1 (1.2%) | 4 (1.6%) | 1 (0.9%) | 5 (2.2%) |  |
|  | yes | 864 (96.8%) | 194 (91.5%) | 85 (98.8%) | 252 (98.4%) | 115 (99.1%) | 218 (97.8%) |  |
|  | missing | 52 | 25 | 0 | 9 | 12 | 6 |  |
|  | ml transfused (median (IQR)) | 3000 (1400-5905) | 1500 (600-2775) | 4375 (2400-8700) | 4500 (2150-8000) | 2700 (1500-4775) | 3300 (1680-6000) | **<0.001** |
| Platelet concentrates | n (%) |  |  |  |  |  |  | **<0.001** |
|  | no | 231 (38.4%) | 71 (57.3%) | 21 (36.2%) | 42 (21.6%) | 31 (49.2%) | 66 (40.5%) |  |
|  | yes | 371 (61.6%) | 53 (42.7%) | 37 (63.8%) | 152 (78.4%) | 32 (50.8%) | 97 (59.5%) |  |
|  | missing | 343 | 113 | 28 | 71 | 65 | 66 |  |
|  | ml transfused (median (IQR)) | 300 (0-1200) | 0 (0-400) | 550 (0-1400) | 800 (200-2300) | 150 (0-1000) | 250 (0-900) | **<0.001** |
| Fresh frozen plasma | n (%) |  |  |  |  |  |  | **<0.001** |
|  | no | 270 (53.9%) | 75 (73.5%) | 19 (40.4%) | 55 (34.2%) | 37 (72.5%) | 84 (60%) |  |
|  | yes | 231 (46.1%) | 27 (26.5%) | 28 (59.6%) | 106 (65.8%) | 14 (27.5%) | 56 (40%) |  |
|  | missing | 444 | 135 | 39 | 104 | 77 | 89 |  |
|  | ml transfused (median (IQR)) | 0 (0-1500) | 0 (0-400) | 500 (0-2200) | 880 (0-2200) | 0 (0-440) | 0 (0-1000) | **<0.001** |
| Prothrombin complex concentrate | n (%) |  |  |  |  |  |  | **<0.001** |
|  | no | 807 (86.2%) | 229 (96.6%) | 65 (77.4 %) | 194 (74.0%) | 114 (90.5%) | 205 (90.3%) |  |
|  | yes | 109 (11.6%) | 6 (2.5%) | 17 (20.2%) | 59 (22.5%) | 7 (5.6%) | 20 (8.8%) |  |
|  | unknown | 20 (2.1%) | 2 (0.8%) | 2 (2.4%) | 9 (3.4%) | 5 (4.0%) | 2 (0.9%) |  |
|  | missing | 9 (1.0%) | 0 (0.0%) | 2 (2.3%) | 3 (1.1%) | 2 (1.6%) | 2 (0.9%) |  |
|  | IU transfused (median (IQR)) | 2500 (2000-3500) | 1750 (1000-3000) | 3000 (2500-6500) | 3000 (2000-4000) | 1500 (1000-4000) | 2750 (1500-5000) | 0.211 |
| Coagulation factor XIII | n (%) |  |  |  |  |  |  | **<0.001** |
|  | no | 575 (61.3%) | 187 (79.2%) | 43 (50.6%) | 119 (45.1%) | 86 (68.3%) | 140 (61.7%) |  |
|  | yes | 342 (36.5%) | 47 (19.9%) | 38 (44.7%) | 134 (50.8%) | 36 (28.6%) | 87 (38.3%) |  |
|  | unknown | 21 (2.2%) | 2 (0.8 %) | 4 (4.7%) | 11 (4.2%) | 4 (3.2%) | 0 (0.0%) |  |
|  | missing | 7 (0.7%) | 1 (0.4%) | 1 (1.2%) | 1 0.4%) | 2 (1.6%) | 2 (0.9%) |  |
|  | IU transfused (median (IQR)) | 3750 (3750-5000) | 2500 (2500-5000) | 2500 (2500-5000) | 3750 (3000-5000) | 3750 (3750-7500) | 3750 (2500-6250) | 0.109 |
| **Laboratory values, day 1** | | | | | | | | |
| Platelets [1000/µl] | median (IQR) | 207.5 (145-277.5) | 225.5 (157.5-284) | 203 (143-266) | 197.5 (136-277.5) | 213.5 (160.5-291.5) | 200 (143-272) | 0.117 |
|  | missing | 29 | 5 | 1 | 13 | 4 | 6 |  |
| aPTT [sec.] | median (IQR) | 44.1 (34.4-58.3) | 43.5 (36-55) | 46 (33-58) | 45 (35-63) | 42 (34-55.35) | 45.3 (33.5-60) | 0.698 |
|  | missing | 27 | 8 | 1 | 12 | 4 | 2 |  |
| Quick [%] | median (IQR) | 78.5 (64-95) | 82 (65-98) | 71 (62-86) | 79 (64-93) | 76 (63-89) | 79 (65-98) | **0.018** |
|  | missing | 63 | 14 | 3 | 22 | 9 | 15 |  |
| INR | median (IQR) | 1.13 (1.03-1.3) | 1.1 (1-1.25) | 1.2 (1.07-1.37) | 1.13 (1.03-1.3) | 1.18 (1.06-1.32) | 1.11 (1-1.27) | **0.012** |
|  | missing | 93 | 23 | 4 | 31 | 13 | 22 |  |
| D-Dimer [mg/l] | median (IQR) | 5.76 (2.7-12.965) | 5.87 (2.441-12.99) | 7.8 (3.61-18.53) | 5.645 (2.84-11.87) | 6.673 (3.425-13.54) | 4.4 (2.48-10.5) | 0.134 |
|  | missing | 305 | 83 | 23 | 83 | 48 | 68 |  |
| Fibrinogen [g/l] | median (IQR) | 5.8 (4-7.35) | 5.75 (4-7.2) | 6.015 (4.15-7.12) | 5.225 (3.7-7.295) | 6.38 (4.9-8.23) | 6.045 (4.5-7.23) | **0.030** |
|  | missing | 239 | 55 | 14 | 61 | 38 | 71 |  |
| Faktor XIII [%] | median (IQR) | 54 (43-69) | 60 (50-75) | 50.2 (40-70) | 50 (42-63) | 46.75 (39-53) | 60 (48-75.5) | **0.031** |
|  | missing | 778 | 214 | 71 | 218 | 110 | 165 |  |
| Antithrombin [%] | median (IQR) | 73 (60-86) | 78 (63-91) | 71.5 (60.5-77) | 73 (60-84) | 70.5 (58.5-80.5) | 74.5 (57.5-88) | 0.097 |
|  | missing | 464 | 127 | 38 | 114 | 68 | 117 |  |

**Table S1**: Comorbidities, adjunct medication and laboratory values are presented in total population and in subgroups no BTE vs. major bleeding plus major thromboembolism vs. only major bleeding vs. only major thromboembolism vs. minor events, respectively. Descriptive statistics are expressed as frequencies for categorical variables (including a category for missing data) and as median (IQR) for continuous variables. Differences between groups were tested using the Kruskal-Wallis-test (continuous variables) or Pearson-χ2-test (categorical variables). P-values less than 0.05 were considered as statistically significant.

**Supplemental Figure 1: Risk factors of bleeding events during ECMO support in subgroups**

**a) Only intracranial bleeding**

**
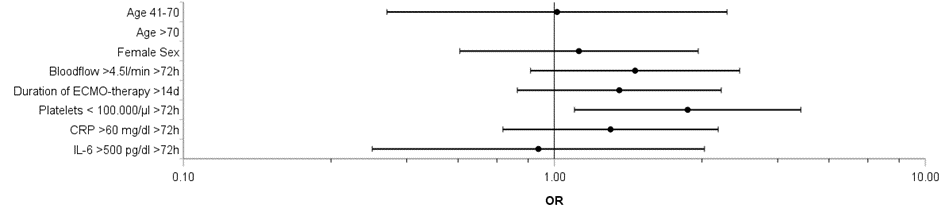
**

**b) Only pulmonary bleeding**

**
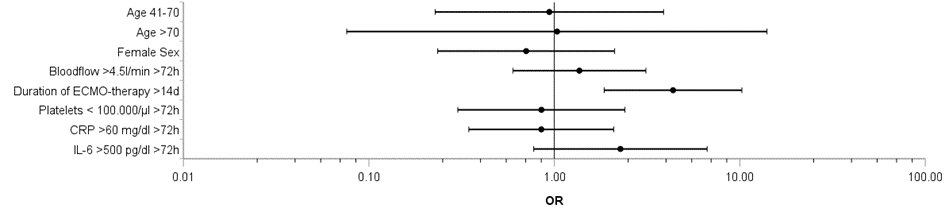
**

**c) Only major intraperitoneal bleeding**

**
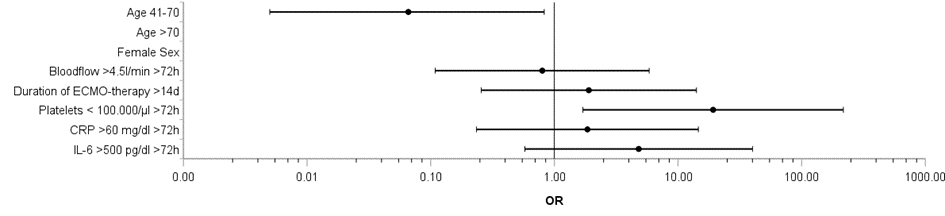
**

**d) Only major bleeding ECMO cannula**

**
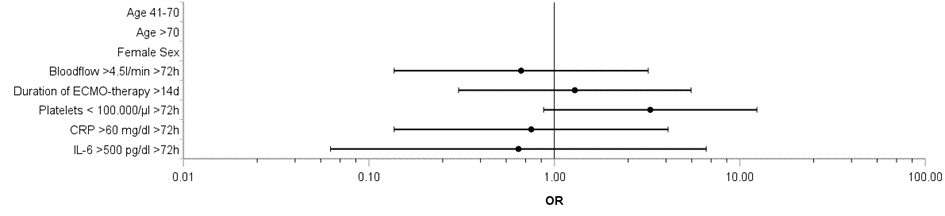
**

**e) Major bleeding other than above**

**
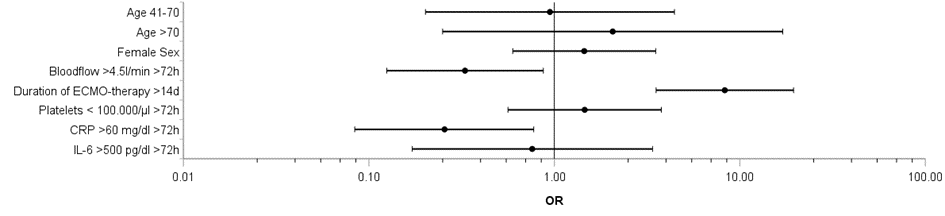
**

**Figure S1**: Subdivisions show risk factors during ECMO support for subgroups **a)** Only Intracranial bleeding **b)** Only pulmonary bleeding **c)** Only major intraperitoneal bleeding **d)** Only major bleeding ECMO cannula **e)** Major bleeding other than above. Variables were selected a priori based on clinical background knowledge: age (41-70 or >70 years vs. 18-40 years), sex (female vs. male), duration of ECMO therapy (>14 days vs. ≤14 days), blood flow (>4.5L/min ≥72h vs. ≤4.5L/min for ≥72h or >4.5L/min for ≤72h), platelet count (<100.000/µL for ≥72h vs. ≥100.000µL for ≥72h or <100.000/µL for <72h), C-reactive protein (CRP) levels (>60mg/dL for ≥72h vs. ≤60mg/dL for ≥72h or >60mg/dL for <72h), interleukin-6 (IL-6) (>500 pg/dL for ≥72h vs. ≤500pg/dL for ≥72h or >500pg/dL for <72h). Analyses were performed by adjusted logistic regression for subgroups a,b,e and univariate χ2 test for subgroups c and d. OR and CI are depicted.

**Supplemental Figure 2: Risk factors of thromboembolic events during ECMO support in subgroups**

**Only Major Pulmonary Embolism**

**
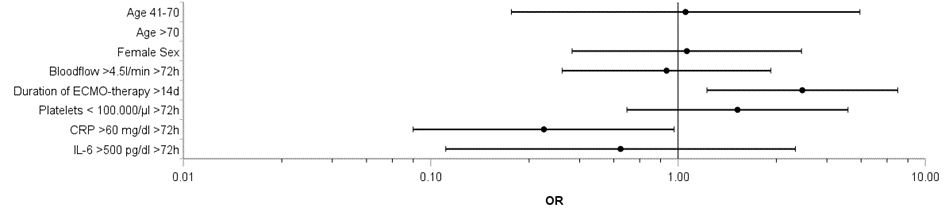
**

**b) Only Major Thromboembolism other than above**

**
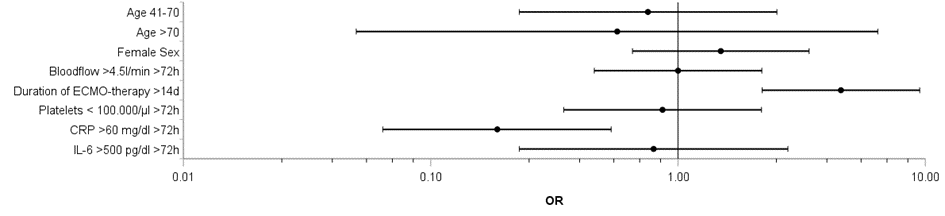
**

**Figure S2**: Subdivisions show risk factors during ECMO support for subgroups **a)** Only major pulmonary embolism **b)** Only major thromboembolism other than above. Variables were selected a priori based on clinical background knowledge: age (41-70 or >70 years vs. 18-40 years), sex (female vs. male), duration of ECMO therapy (> 14 days vs. ≤14 days), blood flow (>4.5L/min ≥72h vs. ≤4.5L/min for ≥72h or >4.5L/min for ≤72h), platelet count (<100.000/µL for ≥72h vs. ≥100.000µL for ≥72h or <100.000/µL for <72h), C-reactive protein (CRP) levels (>60mg/dL for ≥72h vs. ≤60mg/dL for ≥72h or >60mg/dL for <72h), interleukin-6 (IL-6) (>500 pg/dL for ≥72h vs. ≤500pg/dL for ≥72h or >500pg/dL for <72h). Analyses were performed by adjusted logistic regression. OR and CI are depicted.

**Supplemental Figure 3: Sensitivity analysis, risk factors for hemostatic complications during ECMO support in main groups**

**a) Major bleeding plus thromboembolism**


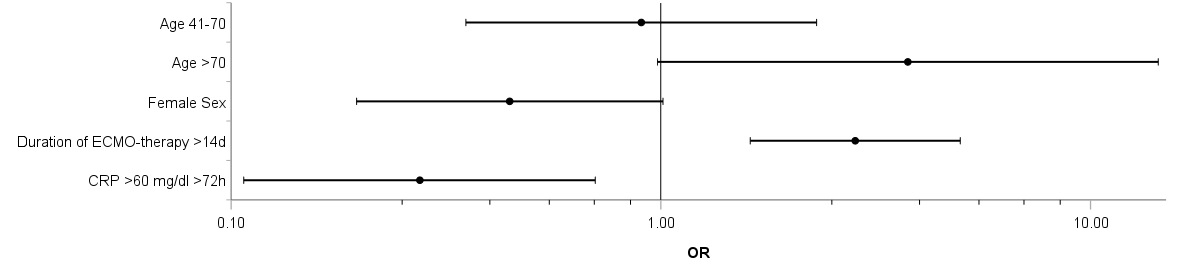


**b) Major bleeding**


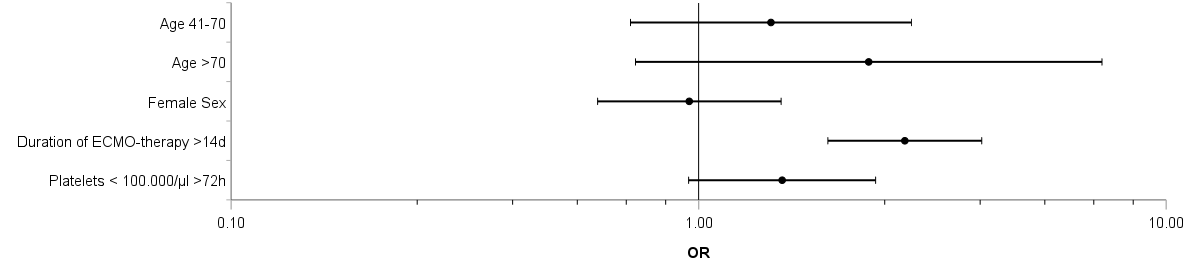


**c) Major thromboembolism**


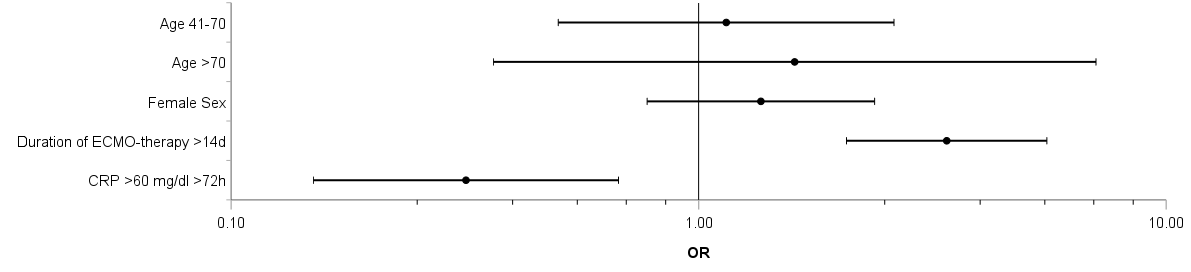


**d) Minor event**


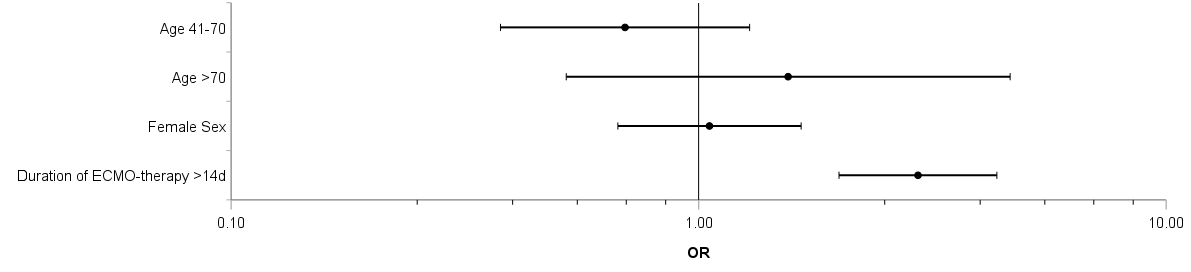


**Figure S3**: Subdivisions show sensitivity analyses for risk factors during ECMO support for the main groups **a)** Major bleeding plus thromboembolism **b)** Major bleeding **c)** Major thromboembolism **d)** Minor events. Variables were selected a priori based on clinical background knowledge: age (41-70 or >70 years vs. 18-40 years), sex (female vs. male), duration of ECMO therapy (>14 days vs. ≤ 14days), platelet count (<100.000/µL for ≥72h vs. ≥100.000µL for ≥72h or <100.000/µL for <72h), C-reactive protein (CRP) levels (>60mg/dL for ≥72h vs. ≤60mg/dL for ≥72h or >60mg/dL for <72h). Analyses were performed by adjusted logistic regression. OR and CI are depicted.

**Supplemental Figure 4: Sensitivity analyses, Risk factors of bleeding events during ECMO support in subgroups**

**a) Only intracranial bleeding**


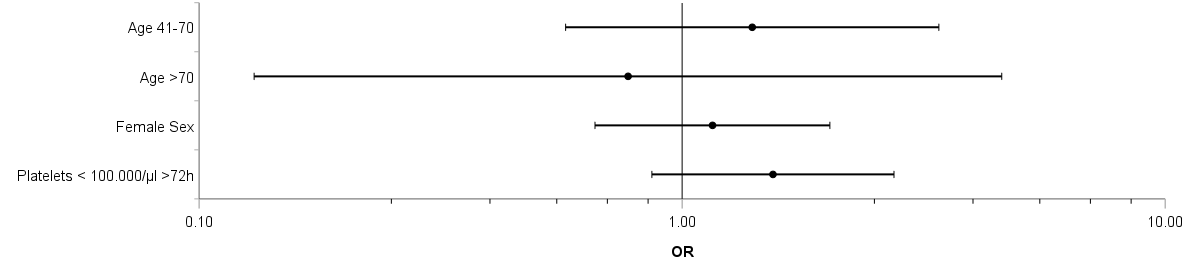


**b) Only pulmonary bleeding**


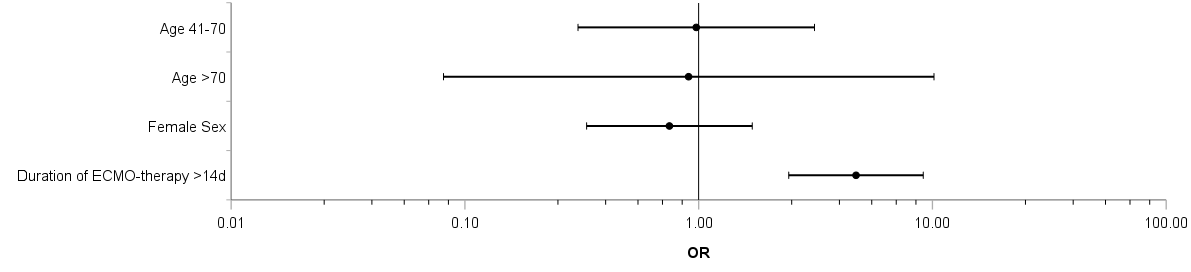


**c) Only major intraperitoneal bleeding**

| **Variable** | **OR (95% CI)** | **p-value** |
| --- | --- | --- |
| Age (41-70) | 0.9 (0.2-4.4) | 0.946 |
| Age (>70) | 1.4 (0.1-18.1) | 0.778 |
| Female Sex | 0.5 (0.2-1.9) | 0.343 |
| Duration of ECMO therapy >14d | 3.0 (1.2-7.4) | 0.014 |
| Blood flow >4.5L/min >72h | 1.3 (0.5-3.6) | 0.575 |
| Platelets <100.000/µL ≥72h | 3.1 (1.2-8.1) | 0.015 |
| CRP >60mg/dL ≥72h | 1.2 (0.4-4.0) | 0.714 |
| IL-6 >500 pg/dL≥72h | 4.3 (0.7-25.7) | 0.081 |

**d) Only major bleeding ECMO Cannula**

| **Variable** | **OR (95% CI)** | **p-value** |
| --- | --- | --- |
| Age (41-70) | n.a. |  |
| Age (>70) | n.a. |  |
| Female Sex | 0.3 (0.1-1.5) | 0.145 |
| Duration of ECMO therapy >14d | 1.1 (0.4-2.9) | 0.810 |
| Blood flow >4.5L/min >72h | 0.5 (0.1-1.8) | 0.298 |
| Platelets <100.000/µL ≥72h | 1.6 (0.5-4.5) | 0.416 |
| CRP >60mg/dL ≥72h | 1.5 (0.5-4.2) | 0.494 |
| IL-6 >500 pg/dL ≥72h | 0.9 (0.1-7.2) | 0.858 |

**e) Major bleeding other than above**


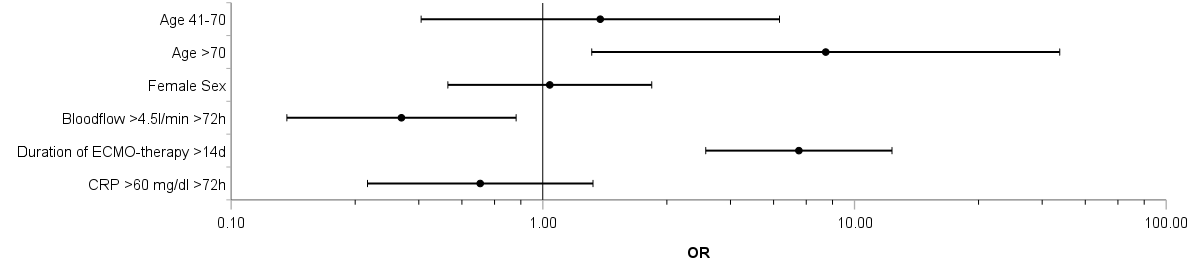


**Figure S4**: Subdivisions show sensitivity analyses for risk factors during ECMO support for subgroups **a)** Only intracranial bleeding **b)** Only pulmonary bleeding **c)** Only major intraperitoneal bleeding **d)** Only Major bleeding ECMO cannula **e)** Major bleeding other than above. Variables were selected a priori based on clinical background knowledge: age (41-70 or >70 years vs. 18-40 years), sex (female vs. male), duration of ECMO therapy (>14 days vs. ≤14 days), blood flow (>4.5L/min ≥72h vs. ≤4.5L/min for ≥72h or >4.5L/min for ≤72h), platelet count (<100.000/µL for ≥72h vs. ≥100.000µL for ≥72h or <100.000/µL for <72h), C-reactive protein (CRP) levels (>60mg/dL for ≥72h vs. ≤60mg/dL for ≥72h or >60mg/dL for <72h). Analyses were performed by adjusted logistic regression for subgroups a,b,e and univariate χ2 test for subgroups c and d. OR and CI are depicted.

**Supplemental Figure 5: Sensitivity analyses, Risk factors of thromboembolic events during ECMO support in subgroups**

**a) Only major pulmonary embolism**


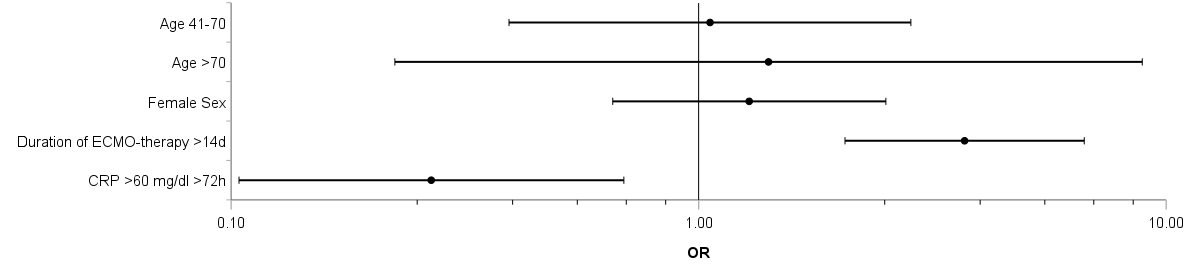


**b) Only major thromboembolism other than above**


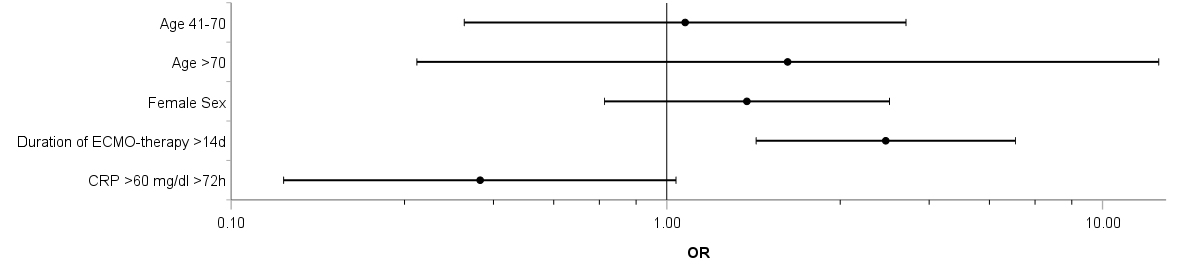


**Figure S5**: Subdivisions show sensitivity analyses for risk factors during ECMO support for subgroups **a)** Only major pulmonary embolism **b)** Only major thromboembolism other than above. Variables were selected a priori based on clinical background knowledge: age (41-70 or >70 years vs. 18-40 years), sex (female vs. male), duration of ECMO therapy (>14 days vs. ≤14 days), blood flow (>4.5L/min ≥72h vs. ≤4.5L/min for ≥72h or >4.5L/min for ≤72h), platelet count (<100.000/µL for ≥72h vs. ≥100.000µL for ≥72h or <100.000/µL for <72h), C-reactive protein (CRP) levels (>60mg/dL for ≥72h vs. ≤60mg/dL for ≥72h or >60mg/dL for <72h), interleukin-6 (IL-6) (>500 pg/dL for ≥72h vs. ≤500pg/dL for ≥72h or >500pg/dL for <72h). Analyses were performed by adjusted logistic regression. OR and CI are depicted.
